# Contrast-induced Thrombosis in Acute Mild Pancreatitis

**DOI:** 10.7759/cureus.4692

**Published:** 2019-05-17

**Authors:** Sindhura Kolli, David Maslak

**Affiliations:** 1 Internal Medicine, The Brooklyn Hospital Center, New York, USA; 2 Internal Medicine, Danbury Hospital, Danbury, USA

**Keywords:** contrast induced thrombosis, pancreatitis, hepatic vein thrombosis, gastroenterology, splenic vein thrombosis

## Abstract

In acute pancreatitis, the most crucial aspect of management falls within the first 48-72 hours, which should be approached in a step-wise order. When steps are skipped or rushed, such as the early use of computed tomography (CT) with contrast in the setting of poor oral intake, the risk of morbidity increases. This is a case when the deleterious effects of contrast worsened the severity of the clinical course, resulting in a higher rate of mortality and longer hospital stay, and escalated the healthcare cost burden.

## Introduction

In acute pancreatitis, the most crucial aspect of management falls within the first 48-72 hours. During this time, life-threatening sequelae can be averted if it is accurately and swiftly diagnosed with the prompt initiation of management. Diagnosis involves the collective presence of clinical symptoms along with radiological evidence via ultrasound or computed tomography (CT) without contrast of the abdomen. Failure of discernible improvement within 72 hours or if classified as severe pancreatitis as per the revised Atlanta classification of pancreatitis, CT with contrast is indicated [1]. However, if contrast CTs are inappropriately or gratuitously utilized, they can catalyze a thrombotic prone environment, resulting in splenic and portal vein thrombosis much like in our patient. Furthermore, the deleterious effects of contrast can worsen the severity of the clinical course, resulting in a higher rate of mortality, longer hospital stay, and escalate the healthcare cost burden, which already attributes $2.6 billion in treating pancreatitis-inflicted patients annually [2-3].

## Case presentation

**Figure 1:**
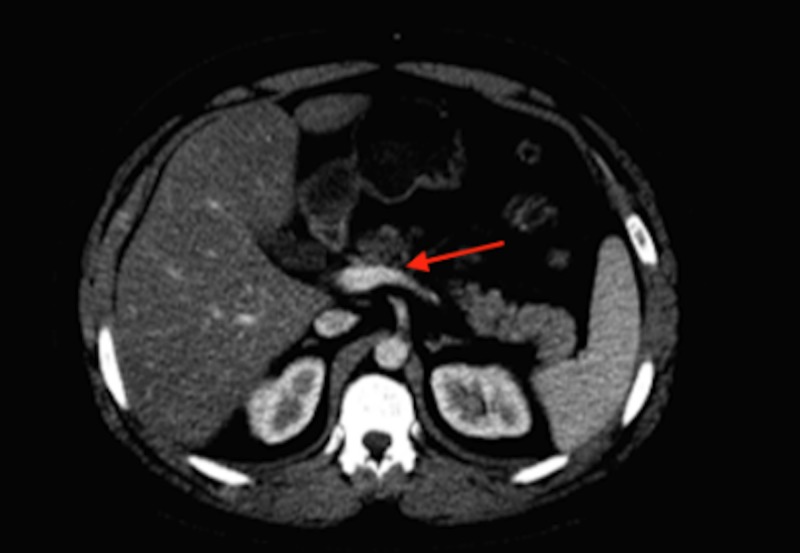
CT abdomen with contrast demonstrating acute pancreatitis

A 48-year-old Caucasian male with a past medical history of hypertension presented to the emergency department (ED) with epigastric abdominal pain, nausea, and vomiting. Vitals were stable. Lipase was elevated to over 600, alanine transaminase was 46, and aspartate transaminase was 41. All other labs were within normal limits. An abdominal computed tomography (CT) with contrast was done immediately, which demonstrated edema of the pancreas and peripancreatic collection of fluid, indicating acute pancreatitis (Figure 1).

**Figure 2:**
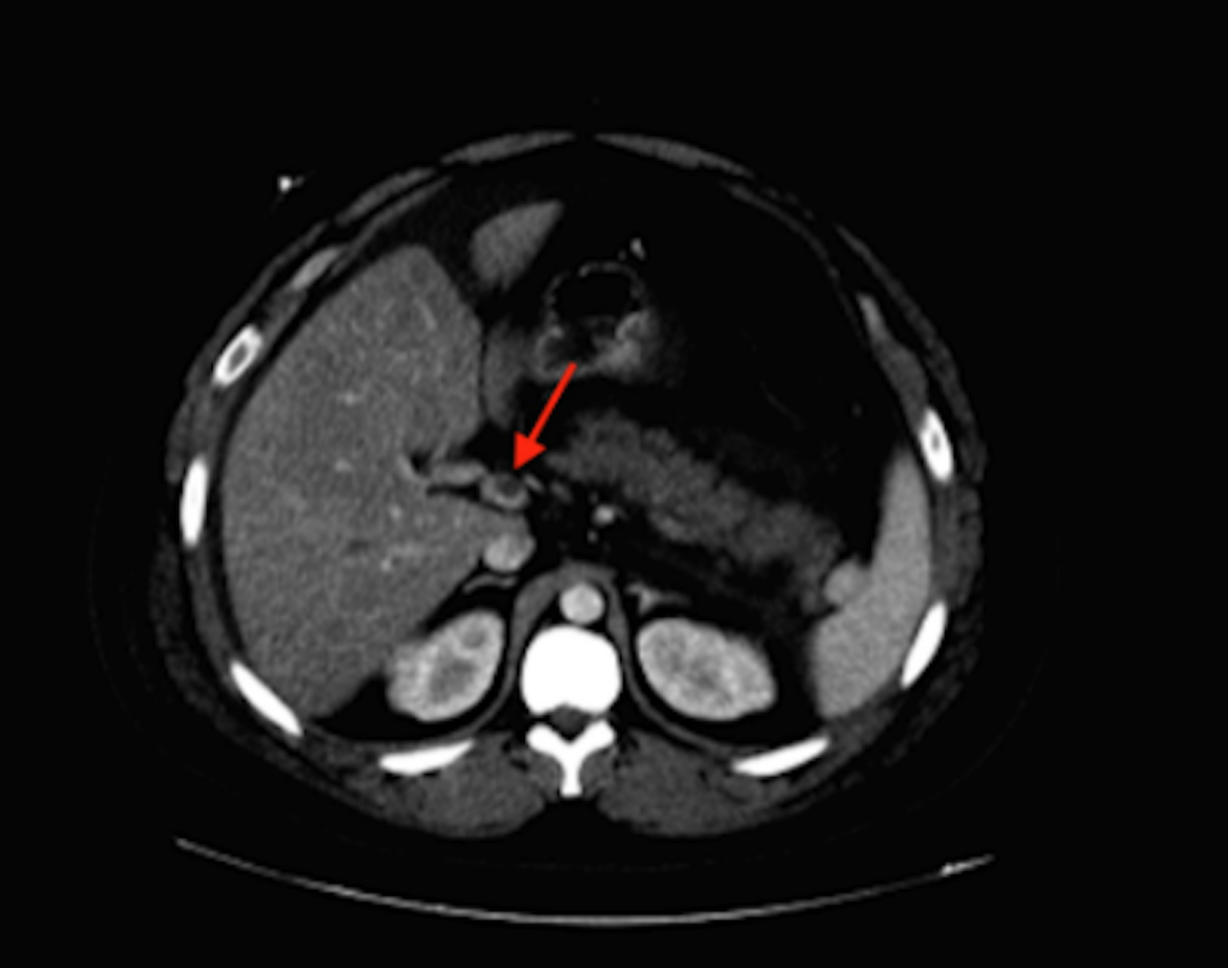
Thrombosis of the hepatic and portal vein in CT abdomen without contrast

Per-oral (PO) pain medication was initiated, as there was difficulty in achieving intravenous (IV) access, so the patient was unable to receive IV fluids. Oral rehydration was encouraged. Due to the intractable uncontrolled pain, the patient was transferred. Upon transfer, the IV line was secured and fluids were started. However, due to the persistence of epigastric pain, a repeat CT abdomen without contrast was performed to ascertain whether the pancreatitis was worsening or if other differentials should be considered. On this CT, thrombosis of the hepatic vein and splenic vein was evident (Figure 2).

The patient was started on a heparin drip while pancreatitis symptoms eventually dissipated with supportive treatment. The patient was transitioned to PO blood thinners and discharged with follow-up.

## Discussion

Approaching pancreatitis within the first 72 hours of admission requires appropriate triage, management, and reassessment based upon the revised Atlanta classification [1]. If the patient is considered to be inflicted with mild or moderate pancreatitis, then an ultrasound of the abdomen along with classical clinical symptoms is appropriate. If the patient worsens or does not improve within this 72-hour window of initiating treatment, or if the patient presents initially as severe pancreatitis, or if necrotizing pancreatitis is suspected, CT of the abdomen with contrast is indicated to assess the extent of necrosis of the solid organ [4]. However, this diagnostic modality comes with its distinct disadvantages.

Intravenous (IV) contrast has led to increased severity of pancreatitis, adversely affecting organ perfusion and microcirculation, leading to the complications of necrosis and infection [2,5-8]. This effect is heightened in those with a body mass index (BMI) of more than 25 [9]. It also can exacerbate an already thrombotic prone environment, resulting in venous thrombosis [10]. In pancreatitis, patient are at a higher risk of thrombosis due to a multitude of factors, such as pancreatic necrosis leaking enzymes, causing irritant vasculitis and subsequent thrombosis, fluid collections, or peri-pancreatic edema compressing vessels with resultant stasis of blood flow, hepatic dysfunction causing deficiencies in protein C and S, hypoalbuminemia, increase in acute phase reactant, fibrinogen, as well as hypovolemia, diminishing organ perfusion and the immobility of the patient. This can result in clot formation most commonly in the splenic vein followed by the portal vein, as well as the splanchnic vein [11-13]. Rarely, it can cause renal vein and inferior vena cava thrombosis [14]. For any acute thrombotic event, immediate anticoagulation must be initiated [11-12]. The use of contrast can also exacerbate acute pancreatitis and increase its severity, resulting in systemic complications [5-8]. As its excretion pathway is via the kidneys, repeated use can impair renal function [10]. These effects culminate in prolonged hospital stays, increased from six to 10 days on average and increased mortality, and portend a poorer prognosis [4,6,9,15]. These complications are more commonly seen in severe cases of pancreatitis, with a higher acute physiological assessment and chronic health evaluation II (APACHE II) scores and higher CT severity index (CTSI) scores where CT with contrast is indicated. The concomitant sepsis does serve as a confounder to completely attributing the thrombotic events, higher mortality rates, longer hospital stays, and increased cost burden to the use of IV contrast alone. However, it does serve as a reminder that IV contrast is warranted only when the aforementioned indicators are present; otherwise, alternatives such as magnetic resonance imaging (MRI) or magnetic resonance cholangiopancreatography (MRCP) should be considered [1,9].

## Conclusions

When patients present with acute pancreatitis, less damaging imaging modalities, such as an ultrasound or CT without contrast, of the abdomen should be considered, especially in patients with difficulty in securing IV access for the administration of fluids. Pancreatitis with concurrent IV contrast leads to a prothrombotic environment, setting the stage for hepatic and portal vein thrombosis like in our patient. This adverse event is why CT of the abdomen with contrast should be reserved if the patient with pancreatitis does not improve within 72 hours of initiating treatment.
